# Minutes of the Ohio Dental College Association

**Published:** 1866-09

**Authors:** J. Taft


### Proceedings of Societies.

### MINUTES OF THE OHIO DENTAL COLLEGE ASSOCIATION.

THE regular annual meeting of the Ohio Dental College Association was held in the lecture room of the College, on Tuesday, February 20th, 1866, at 11 o'clock A. M.

The President, Dr. J. G. Cameron, called the meeting to order.

The following members were present: Doctors A. Berry, J. G. Cameron, Geo. H. Cushing, H. A. Smith, M. DeCamp, H. R. Smith, W. W. Allport, James Taylor, C. W. Spalding, James Leslie, H. J. McKellops, Geo. Watt, C. B. Chapman, W. H. Morgan, A. S. Talbert, G. W. Redman, W. H. Shadoan, S. N. Rhoades, A. A. Blount, J. Taft, Geo. W. Keely, J. P. Ulery, B. D. Wheeler.

The minutes of the last meeting were read.

The Committee on the Amendment of the Charter made the following report:

Your committee to whom was referred the work of securing an amendment to the charter of this institution from the Legislature of the State of Ohio would report that they have accomplished the work assigned them—securing such an amendment to the charter as was indicated at the last annual meeting of this body. It is as follows, viz:

### CHARTER.

### A BILL

TO REGULATE COLLEGES OF DENTAL SURGERY.

SECTION 1. "*Be it enacted by the General Assembly of the State of Ohio.* That the Board of Trustees of any College of Dental Surgery heretofore incorporated and organized under any law of this State shall consist of nine members, and shall be elected by the stockholders of such College in a manner hereinafter provided.

SEC. 2. "The stockholders of such College of Dental Surgery shall, at their first annual meeting after the passage of this act, proceed to elect nine Trustees of such College, three of whom to serve as such for two years, and three of whom to serve as such for three years, and annually thereafter shall elect three Trustees to serve for the term of three years; *Provided*, that vacancies in said Board of Trustees occurring from any other cause than expiration of the time of the Trustee creating such vacancy shall be filled by the election by said stockholders of a Trustee or Trustees to serve only for the unexpired part of such term.

SEC. 3. "This act shall be in force from and after its passage."

J. TAFT,
J. TAYLOR,
W. P. HORTON,
*Committee.*

On motion,

*Resolved*, That the election of a Board of Trustees be the special order of business for Thursday morning at 9 o'clock.

The following persons having become stockholders, their names were proposed for membership in this association, viz: W. W. Sheffield, New London, Conn.; J. C. Dean, Chicago, Ills.; H. S. Chase, Iowa City, Iowa; S. J. Cobb, Nashville, Tenn.; W. H. Morgan, Nashville, Tenn.; A. A. Blount, Springfield, O., and W. H. Shadoan, Louisville, Ky. Upon balloting, all were found to be unanimously elected.

Dr. Allport moved that a committee of three be appointed to nominate candidates for officers for the ensuing year.

Drs. Allport, Spalding and Berry were appointed that committee.

It was moved that a committee of three be appointed to nominate candidates for a Board of Trustees.

That committee was constituted by the appointment of Drs. Geo. H. Cushing, H. J. McKellops and H. A. Smith.

The committee for the nomination of officers reported as follows:

President—H. J. McKellops.

First Vice-President—H. A. Smith.

Second Vice-President—J. C. Dean.

Secretary—J. Taft.

Treasurer—J. G. Cameron.

Upon balloting, the nominations were all confirmed.

The following bill was presented and ordered to be paid:

*Ohio Dental College Association,*

To J. Taft, *Dr.*

To expenses incurred in the procurement of the amendment to the charter $25 00

Cincinnati, February 20th, 1866.

Adjourned till 2 o'clock P. M.

First Day—*Afternoon Session.*

Association met according to adjournment, President Dr. Cameron in the Chair.

Minutes of the morning session were read and approved.

The time having arrived for installing the newly-chosen officers, Drs. Spalding and Allport were appointed a committee to conduct the President elect to the Chair; upon taking which he expressed a just appreciation of the honor conferred upon him, and solicited the cooperation of all the members present in the accomplishment of the work which laid before them.

The Treasurer made the following report:

"The Treasurer would respectfully submit the following:

James Taylor, in account with Ohio Dental College Association, *Dr.*

To cash received on stock, since last report, of

| | |
|---|---|
| A. Blake | $100 00 |
| J. G. Cameron | 100 00 |
| B. D. Wheeler | 50 00 |
| N. B. Slayton | 100 00 |
| L. D. Walter | 20 00 |
| S. J. Cobb | 100 00 |
| J. Taft | 100 00 |

| | |
|---|---|
| J. C. Dean | 100 00 |
| W. W. Allport | 60 00 |
| H. A. Smith | 100 00 |
| H. R. Smith | 57 53 |
| Geo. E. Jones | 57 53 |
| J. Chesebrough | 36 32 |
| Balance on hand at last report | 173 91 |
| | 1,155 59 |

Drs. Spalding and Cushing were appointed an auditing committee, who having examined the above account, reported it to be correct.

Dr. Taylor made some explanatory remarks in regard to unpaid stock, in which he presented the fact that a small amount was yet unprovided for to liquidate the entire indebtedness upon the institution.

The President appointed the following gentlemen as collectors of stock due at the places named:

J. B. Dunlevy, Pittsburg, Pa.

George H. Cushing, Chicago, Ills.

A. M. Leslie, St. Louis, Mo.

W. P. Horton, Cleveland, O.

A. Berry, Cincinnati, O.

The Faculty presented the following report, viz:

REPORT OF THE FACULTY OF THE OHIO COLLEGE OF DENTAL SURGERY FOR THE SESSION ENDING FEBRUARY 21, 1866.

*To the President of the Ohio Dental College Association:*

The Faculty of the Ohio College of Dental Surgery respectfully report that as early as practicable after the last annual meeting of this Association, the members of the Faculty met and organized by electing Dr. J. Taft Dean.

The Annual Announcement was issued, and such other measures were adopted as were thought necessary to the efficiency of the coming session. These included the appointment of three demonstrators respectively for the departments of Anatomy, Operative and Mechanical Dentistry, the selections being made by the professors of these departments.

Two of the demonstrators, we believe, discharged the duties assigned them with faithfulness and energy, thus adding much to the thoroughness of the course. The other, in response to the call of other duties, we suppose, left the city during the session, and the professor of the department (Mechanical Dentistry) attended to the demonstrations as well as the lectures.

The session began the first Monday of November, a preliminary course having been given from the middle of October. The Professor of Chemistry was unable to lecture at the beginning of the session, and a private arrangement was made by which the class was able, for a few weeks, to hear the chemical lectures of the Miami Medical College, which arrangement, we hope, has given reasonable satisfaction to those interested. The other professors were able to complete their full courses without interruption.

We have the satisfaction to report a larger class than was ever present before in the institution, the number of matriculants being forty-one.

The graduating class is also larger than usual, the number being nineteen, and their names as follows:

P. J. Collins, W. H Pabodie, Miss L. B. Hobbs, H. Newington, George L. Shepard, R. C. Morgan, J. P. Holmes, F. C. Eddelman, W. H. Woodward, A. A. Blount, R. A. Alloway, M. Stout, D. A. Wilder, C. C. Chittenden, A. E. Lyman, J. Nelson, G. W. DeCamp, Geo. P. Ketchum, N. B. Stump.

It will be observed that among these is the name of a lady, which is worthy of notice, as, we believe, the first lady graduate of a Dental College.

The opportunities for clinical instruction during the session have been good.

It is with pleasure we bear testimony to the urbanity and studious habits of the class. The improvement has been manifest, both with the graduates and juniors, and we feel free to state that the class of this session may be regarded as a valuable accession to our ranks, both on account of their correct professional attainments and their extended knowledge of general and collateral sciences. In reference to the latter, they probably surpass any preceding class of the institution. This is as it should be; for, with increased educational facilities, we have a right to expect and demand higher attainments.

Contributions from various members of the profession were made during the year, for improvements of the College building, and for appliances in the Infirmary and Mechanical Laboratory. These, amounting to $503, have been carefully expended, and in addition a sum of $200, appropriated by the Faculty, has been spent in the same way.

As dental collegiate education is of but recent origin, and as there are in the profession many worthy, active, scientific and progressive men, who from age, family ties, or other circumstances beyond their control, are so tied down as to render it impracticable for them to attend a course of lectures, the Faculty has thought proper to recommend a number of such to the Board of Trustees for honorary degrees, and we are happy to state that the Board have cordially carried out our recommendations. The names of the Honorary Graduates are as follows:

C. R. Taft, J. Armstrong, S. B. Smith, B. F. Robinson, F. S. Slosson, B. T. Spelman, C. Palmer, A. W. Maxwell, S. M. Rhoades, J. B. Beauman, C. H. Harroun, R. McDowell, S. W. McDowell, W. H. Shadoan, E. L. Clark, J. A. Robinson, C. N. Woodward.

The Faculty recommend a lengthening of the session, so as to have eight weeks of lectures previous to the holiday vacation, and to have the commencement exercises on the first Wednesday in March. We also suggest that the title of the Chair of "INSTITUTES OF DENTAL SCIENCE" be changed to SPECIAL HISTOLOGY, PHYSIOLOGY AND PATHOLOGY.

Some additional chairs and other appliances are needed in the Infirmary, some lathes and accompaniments in the Mechanical Laboratory, and a supply of apparatus for the chemical department, as well as miscellaneous appliances for general illustration. With these and a relief from its indebtedness, we think a prosperous and useful career awaits your institution.

Respectfully submitted by the Faculty.

On motion, the report was accepted.

The changes recommended in the report of the Faculty were, on motion, adopted.

Remarks were made by various members of the Association, in regard to the practical departments of the College.

Various suggestions were made, with a view of attaining improvement and progress.

*Resolved*, That the Board of Trustees be requested to procure the services of an efficient demonstrator, or demonstrators, for these departments.

Adjourned to 9 o'clock A. M. Thursday next.

SECOND DAY—*Morning Session.*

THURSDAY MORNING.

The Association met according to adjournment.

The minutes of the last session were read and approved.

On motion,

*Resolved*, That this Association hereby accept the amendment to the charter of the Ohio College of Dental Surgery, recently enacted by the Legislature of the State of Ohio.

The Nominating Committee having presented a list of names from which to select nine Trustees for this institution, as provided for by the amended charter, the election of the Board was now declared to be in order.

At this juncture some inquiry was made in regard to the manner of voting for the Trustees. It was decided that proxy votes should not be permitted.

Upon balloting, the following persons were found to be elected, viz:

W. W. Allport, H. A. Smith,
G. W. Keely, H. J. McKellops,
James Taylor, A. Berry,
A. S. Talbert, B. D. Wheeler,
W. H. Morgan.

On motion,

*Resolved*, That a committee of three be appointed to revise the Constitution.

Drs. C. W. Spalding, James Taylor and J. Taft.

The committee appointed two years ago to solicit funds, appliances, specimens, &c., were called upon for a report.

They reported that in money about $500 had been contributed, as well as quite a variety of appliances and instruments, and among these are

A dental chair and footstool, worth $100, contributed by R. Archer, of Rochester, N. Y.

Also, a chair and footstool, by J. S. Walters & Co., of Cincinnati, worth $60.

Two Buckeye Vulcanizers, by Wardle & James, worth $25.

Also, dental goods, by James Leslie, $25.

Dr. McKellops also reported that he had received a contribution from Dr. A. Preterre, of Paris, France—a number of beautiful models, representing various deformities of the mouth, such as cleft palate, fractures and absence of the maxillaries, absence of the tongue, together with the methods of, and appliances for, remedying these defects.

These appliances were brought before the Association, exhibited and fully explained by Dr. McKellops, who had seen nearly all the cases represented by these models, and he gave testimony to the very great efficiency of all these appliances.

On motion,

*Resolved*, That the thanks of this Association are due, and are hereby tendered to Dr. A. Preterre, of Paris, France, for the beautiful models, lately received from him, of six very interesting cases occurring in his practice, and successfully treated by him. These cases comprise three of congenital cleft palate, two of which are very remarkable, the model of the third showing the method by which Dr. P. remedied the defect by means of an inflexible obturator.

One of the three remaining models exhibits a case of malocclusion of the jaws, another the loss of the tongue, and the third the loss of a portion of one of the alæ of the nose.

These models serve to demonstrate the progress which the science and art of dentistry is making in the old world.

2d. *Resolved*, That the Secretary of this Association be requested to furnish Dr. Preterre with a copy of these resolutions.

A vote of thanks was also tendered to all those who have made contributions to this institution.

On motion, the following persons were appointed to receive contributions of preparations, &c., &c., for the College, viz:

Drs. A. Berry, of Cincinnati, Chairman; J. C. Dean, of Chicago; A. S. Talbert, of Lexington, Ky.; H. J. MeKellops, of St. Louis, Mo.; J. B. Dunlevy, of Pittsburg; Jas. Knapp, of New Orleans; W. W. Allport, of Chicago; A. M. Leslie, of St. Louis; M. DeCamp, of Mansfield, O.; W. H. Shadoan, of Louisville, Ky.; A. A. Blount, of Springfield, O.; W. H. Morgan, of Nashville; N. B. Slayton, of Florence, Italy; W. P. Horton, of Cleveland, O.

On motion, proceeded to appoint delegates to the American Dental Association, to meet in Boston July next.

The following were chosen:

Drs. A. S. Talbert, W. H. Shadoan, H. R. Smith, J. P. Ulrey, Geo. W. Keely, A. A. Blount, M. DeCamp, J. Chesebrough, G. W. Redman, James Taylor, W. W. Allport, J. A. McClelland, B. D. Wheeler.

Adjourned to meet on the first Tuesday of March, 1867, 10 o'clock A. M.

J. TAFT, Secretary.

